# Nevirapine Plasma Concentrations Are Associated with Virologic Response and Hepatotoxicity in Chinese Patients with HIV Infection

**DOI:** 10.1371/journal.pone.0026739

**Published:** 2011-10-31

**Authors:** Jia Wang, Huijuan Kou, Qiang Fu, Yang Han, Zhifeng Qiu, Lingyan Zuo, Yanling Li, Zhu Zhu, Min Ye, Qing Ma, Taisheng Li

**Affiliations:** 1 Department of Infectious Disease, Peking Union Medical College Hospital, Chinese Academy of Medical Sciences, Beijing, China; 2 Department of Anesthesiology, Beijing Jishuitan Hospital, Beijing, China; 3 Department of Pharmacy and Pharmacology, Peking Union Medical College Hospital, Chinese Academy of Medical Sciences, Beijing, China; 4 Department of Pharmacy Practice, School of Pharmacy and Pharmaceutical Sciences, University at Buffalo, Buffalo, New York, United States of America; 5 Center for Human Experimental Therapeutics, University of Rochester, Rochester, New York, United States of America; University of Rochester, United States of America

## Abstract

**Background:**

Limited information is available on the relationship between nevirapine plasma concentrations and virologic response or liver toxicity in Chinese patients with HIV infection. The objective of this prospective study was to test this relationship and to determine the minimal therapeutic trough concentration of nevirapine for Chinese patients.

**Methods:**

A total of 227 HIV-infected, treatment naïve patients were enrolled into this study. Blood samples were taken at C_trough_ (12 hr postdose) and C_2_ (2 hr postdose) for measurement of nevirapine concentrations 6 months after treatment initiation. Therapeutic outcomes, viral load and CD4 cell count, were assessed at 3 and 6 months after starting therapy, while the evaluation of hepatotoxicity was undertaken 12 months after nevirapine treatment.

**Results:**

A significant correlation between nevirapine trough concentrations and viral load was noticed after 6 months of treatment, particularly in patients with partial response and viral failure (p<0.01). The therapeutic C_trough_ of nevirapine for Chinese patients was determined to be 3.9 µg/ml using the receiver operating characteristic curve. Virologic failure was observed in 21% (6/29) of patients with low nevirapine concentrations (<3.9 µg/ml) versus 5% (4/87) in patients with concentrations higher than 3.9 µg/ml (p = 0.015). Hepatotoxicity was significantly associated with the median nevirapine trough concentrations among male patients (8.20 *vs.* 5.48 µg/ml, p = 0.015) and hepatitis C virus co-infection (p = 0.039).

**Conclusions:**

Among Chinese patients with HIV infection, the therapeutic C_trough_ of nevirapine was 3.9 µg/ml, higher than the recommended 3.0 µg/ml. The correlation between nevirapine concentrations, efficacy and hepatotoxicity suggests the benefit of dosage adjustment based on therapeutic drug monitoring among Chinese HIV-infected patients to optimize nevirapine containing antiretroviral therapy.

## Introduction

Highly active antiretroviral therapy is considered an effective approach for the management of HIV/AIDS. Due to its low cost and high efficacy, nevirapine -containing regimens are often preferable to others in resource-limited countries [1]. Approximately 80% Chinese patients receiving antiretroviral therapy are currently on nevirapine-containing regimens. The pharmacological characteristics of nevirapine make it an attractive candidate for therapeutic drug monitoring, as previous studies have indicated a significant relationship between nevirapine trough concentrations and virologic response [2], [3], [4]. Also, high concentrations are linked to an increased risk of liver toxicity and hepatic injury in patients with chronic hepatitis C [5], [6], [7], [8]. Inter-individual variability of nevirapine plasma concentrations among HIV-infected adults was common in routine clinical practice (∼50%) [9]. The variability could be partially explained by the differences in ethnicity, gender, polymorphisms in enzyme and transporter genes, hepatitis virus co-infection, and concomitant medications [10], [11], [12], [13].

As the majority of the previous studies were performed among Caucasian subjects, there is generally a lack of information regarding the efficacy and safety of nevirapine in Chinese HIV-infected patients. A number of studies exploring the association of exposure to nevirapine with virologic response yielded a target trough concentration of ∼3.0 mg/l [2], [4]; however, this value has not been confirmed among Chinese patients. In addition, the relationship between liver toxicity and nevirapine concentrations has not been established conclusively, though several studies indicate transaminase elevations are related to nevirapine exposure [6], [8]. Therefore, the overall objective of the present study was to investigate the relationship between nevirapine concentrations, virologic response and liver toxicity in Chinese HIV-infected patients.

## Results

### A. Virologic Response

**Figure 1:**
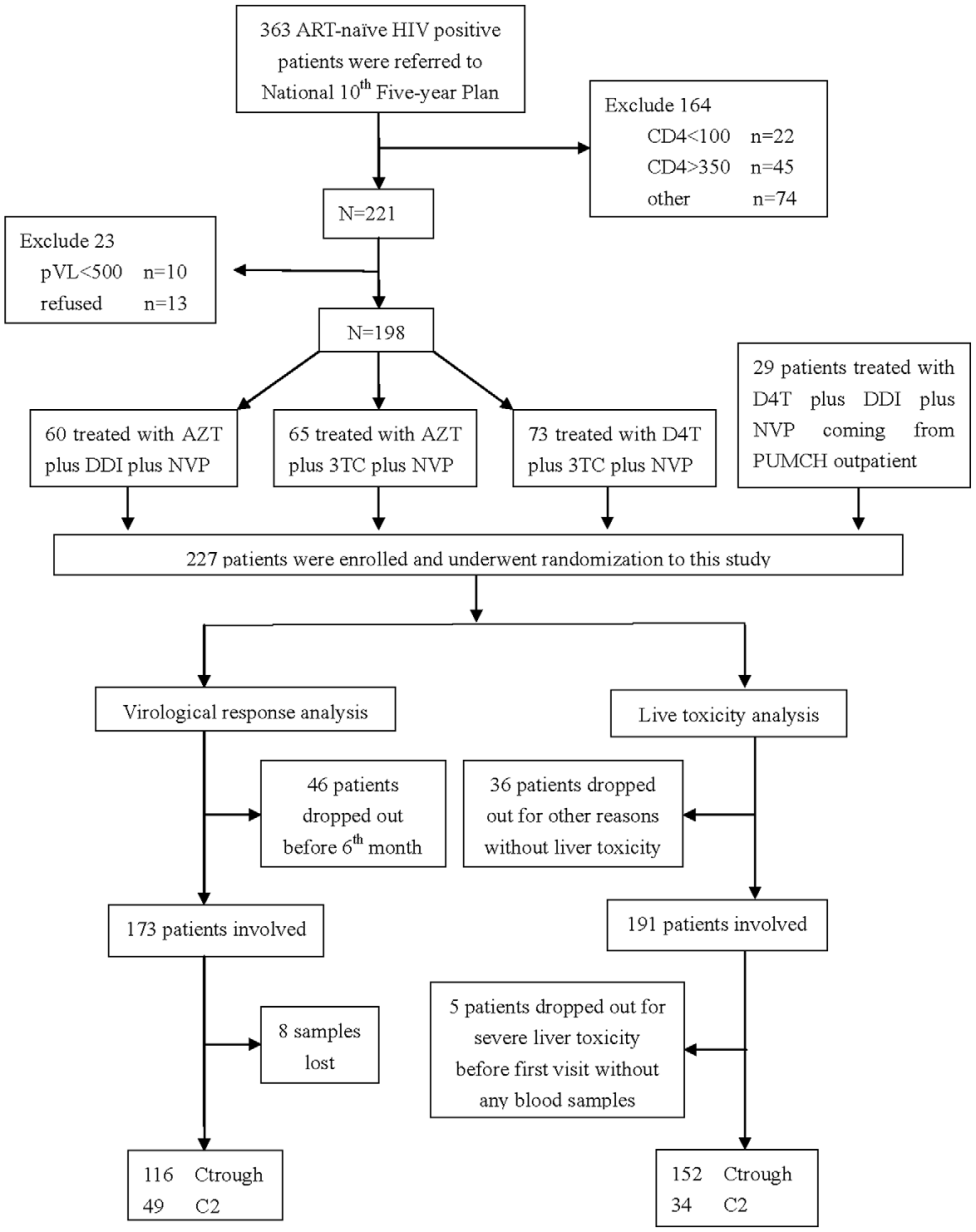
Flow of patient participation through the clinical trial. ART: antiretroviral therapy; VL: viral load; AZT: zidovudine; DDI: didanosine; NVP: nevirapine; 3TC: lamivudine; D4T: stavudine; C_trough_: trough concentrations of nevirapine; C_2_: nevirapine concentrations 2-h post-dose.

Of 227 HIV-positive antiretroviral-naive patients screened, 173 were treated by a fixed combination antiretroviral therapy containing nevirapine for at least 24 weeks and included in the virologic response analysis (Figure 1). Demographic characteristics of these patients are summarized in Table 1. There were no statistically significant differences among the three groups (complete response, partial response and viral failure) at baseline. Plasma nevirapine concentrations were measured 24 weeks after treatment initiation, including C_trough_ (n = 116) and C_2_ (n = 49).

**Table 1:** Baseline Characteristics of Patients Included in Virologic Response Analysis.

	Total n = 173	Complete Response (<50 copies/ml) n = 126	Partial Response (50–400 copies/ml) n = 32	Virologic Failure (>400 copies/ml) n = 15	*p*
Male -n (%)	90 (52)	64 (51)	18 (56)	8 (53)	0.854
Transmission route -n (%)					0.674
Blood transfusion	50 (29)	37 (29)	9 (28)	4 (27)	
Sexual	82 (47)	56 (44)	17 (53)	9 (60)	
Others	41 (24)	33 (26)	6 (19)	2 (13)	
Co-infection -n (%)					
Hepatitis B virus	12 (7)	7 (6)	3 (9)	2 (13)	0.445
Hepatitis C virus	44 (25)	34 (27)	6 (19)	4 (27)	0.630
Age (yrs) -median (IQR)	37 (30, 45)	37 (29, 43)	37 (32, 50)	35 (27, 46)	0.343
Weight (kg) -median (IQR)	56 (50, 65)	56 (50, 67)	55 (49, 60)	60 (53, 70)	0.152
CD4 cell count (cells/L) - mean ± SD	203±91	204±86	197±108	207±98	0.930
HIV-1 RNA (log10 copies) - mean ± SD	4.53±0.70	4.46±0.67	4.62±0.77	4.89±0.69	0.071

Three patient groups, complete response with viral load <50 copies/ml, partial response with viral load 50–400 copies/ml and virologic failure with viral load greater than 400 copies/ml, were included for analysis.

**Figure 2:**
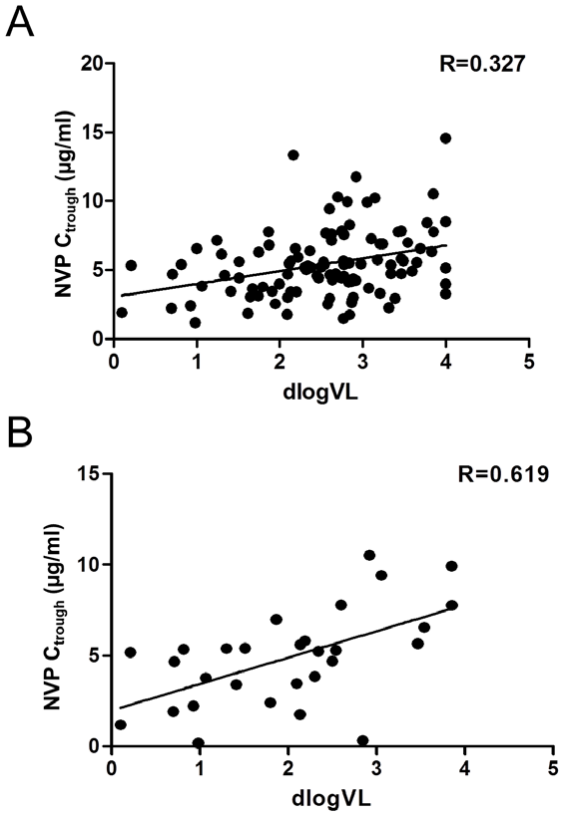
Linear regression analysis of decrease in viral load (VL) and trough concentrations of nevirapine (NVP C_trough_). The log transformation of decrease in viral load (dlogVL) was significantly correlated with trough nevirapine concentrations (NVP Ctrough, µg/ml) among (A) all patients (r = 0.327, *p*<0.05); and (B) patients with partial response (VL 50–400 copies/ml) and virologic failure (VL>400 copies/ml) (r = 0.619, *p*<0.01).

The overall incidence of virologic failure was 10% (18/173). Nevirapine C_trough_ and C_2_ in the three groups are summarized in Table 2. The median C_trough_ in patients with virologic failure was significantly lower than that in partial response and complete response groups (3.81 vs. 5.65 and 5.18 µg/ml, respectively, *p = 0.018*), whereas no significant differences were noted between partial and complete response groups. In contrast, no significant difference in median C_2_ was noted among these groups (*p = 0.608*). A relatively large inter-patient variability in nevirapine C_trough_ was noted (Figure 2, Table 2). Linear regression analysis indicated a significant linear relationship between viral load and C_trough_ (r = 0.327, *p<0.05*) for all patients, particularly when limited to patients with a partial response or viral failure (r = 0.619, *P<0.01*).

**Table 2:** Relationship between Plasma Nevirapine Concentrations [Median (IQR), µg/ml] and Virologic Responses.

	Complete Response (<50 copies/ml)	Partial Response (50–400 copies/ml)	Virologic Failure (>400 copies/ml)	*p*
C_trough_	5.18 (3.97, 7.16) (n = 87)	5.65 (4.69, 7.78) (n = 19)	3.81 (2.14, 5.36) (n = 10)	0.018
C_2_	6.85 (5.47, 9.16) (n = 34)	6.91 (3.93, 8.21) (n = 11)	6.49 (4.82, 8.19) (n = 4)	0.608

Ctrough: trough concentrations; C2: concentrations at 2-h post-dose. Three patient groups, complete response with viral load <50 copies/ml, partial response with viral load 50–400 copies/ml and virologic failure with viral load greater than 400 copies/ml, were included for analysis.

**Figure 3:**
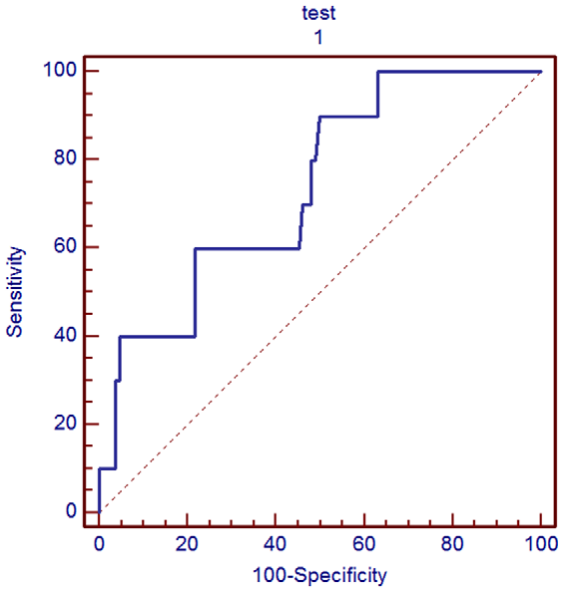
Evaluation of nevirapine trough concentrations as a prognostic factor for virologic failure using ROC curve. The receiver operating characteristic (ROC) curve was utilized to evaluate nevirapine trough concentrations as a prognostic factor for virologic failure accommodating both sensitivity and specificity. A cut-off value of 3.9 µg/ml was determined for Chinese HIV+ patients with a significantly improved sensitivity of 60% in comparison to the original cut-off of 3.0 µg/ml (*p*<0.05, Table 3).

In order to determine the nevirapine C_trough_ threshold for therapeutic drug monitoring in Chinese HIV-infected patients, the receiver operating characteristic curve (ROC curve, Figure 3) was used to evaluate the sensitivities and specificities of different cut-off values as shown in Table 3. While the recommended threshold of 3.0 µg/ml corresponded to a low sensitivity of 40% and a high specificity of 88% for viral failure, a new threshold value of 3.9 µg/ml would have a significantly increased sensitivity of 60% (*p = 0.005*) and an acceptable specificity of 78% (*p = 0.06*). Virologic failure was observed in 21% of patients with concentrations <3.9 µg/ml versus 5% with concentrations higher than 3.9 µg/ml (p = 0.015) (Table 4).

**Table 3:** Sensitivity and Specificity of Cut-Off Values of Nevirapine Trough Concentrations Based on Receiver Operating Characteristic (ROC) Curve Analysis.

C_trough_ (µg/ml)	Sensitivity (%)	Specificity (%)
2.4	40	95
3.0*	40	88
3.4	40	83
3.8	50	78
3.9**	60	78
4.0	60	76
5.2	70	54
5.4	80	52
5.6	90	50
6.2	100	37

*Currently used in clinical practice for Chinese HIV+ patients,

**New therapeutic nevirapine C_trough_ based on the data from this study.

**Table 4:** Risk of Viral Failure in Chinese HIV+ Patients Based on Nevirapine Trough Concentration of 3.9 µg/ml.

	Virologic Response - n (%)		*p*
	Complete and partial response	Virologic Failure	
<3.9	23 (79)	6 (21)	
≥3.9	83 (95)	4 (5)	0.015

Two patient groups, complete and partial response with viral load <50 copies/ml and 50–400 copies/ml, respectively and virologic failure with viral load greater than 400 copies/ml, were included for analysis.

### B. Nevirapine Related Liver Toxicity

Of the 227 patients screened, 191 patients were qualified for the liver toxicity analysis (Figure 1). The incidence of liver toxicity was 71% (136/191), including 43 patients (23%) with severe liver toxicity within 12 weeks of starting therapy. Demographic characteristics of those with and without severe liver toxicity are summarized in Table 5. There were no statistically significant differences between these groups at baseline, except for the body weight.

**Table 5:** Baseline Characteristics of Patients Included in the Liver Toxicity Analysis.

	Total n = 191	Severe Hepatotoxicity n = 42	Without Severe Hepatotoxicity n = 149	*p*
Male -n (%)	94 (49)	19 (45)	75 (50)	0.342
Transmission route -n (%)				0.224
Blood transfusion	57(30)	12(29)	46(31)	
Sexual	95(50)	25(58)	70(47)	
Others	38(20)	5(12)	33(22)	
Co-infection -n (%)				
Hepatitis C virus	56(29)	16(38)	40(27)	0.112
Hepatitis B virus	11(6)	0(0)	11(7)	0.060
Age (yrs) -median (IQR)	37(29, 43)	35(29, 43)	37(29, 43)	0.683
Weight (kg) -median (IQR)	56(50, 64)	55(50, 61)	56(52, 65)	0.020
ALT (U/L) -median (IQR)	27(16, 48)	29(18, 48)	29(17, 49)	0.207
AST (U/L) -median (IQR)	29(22, 43)	29(22, 48)	31(22, 43)	0.578
Tbil (µmol/L) -median (IQR)	11(8, 15)	12(8, 16)	11(8, 15)	0.764
CD4 cell count (cells/L) - mean±SD	210±90	209±98	211±88	0.668
HIV-1 RNA (log10 copies) - mean±SD	4.49±0.69	4.39±0.74	4.51±0.67	0.523

ALT: alanine aminotransferase; AST: aspartate aminotransferase; Tbil: total bilirubin. Two patient groups, with and without severe hepatotoxicity, were included for analysis.

Nevirapine C_trough_ and C_2_ in different groups are summarized in Table 6. There was no significant difference of C_trough_ between these groups (median 5.91 *vs.* 5.54 µg/ml, *p = 0.290*). Previous studies suggest that gender, hepatitis B or C virus co-infection and baseline liver function are independent risk factors for nevirapine related liver toxicity. Thus, the impact of gender, baseline liver function and presence of hepatitis C virus antibody on C_trough_ were evaluated. A similar analysis for C2 was not possible due to the small number of C2 samples. A significantly higher median C_trough_ was noted in males with severe liver toxicity in comparison to those without severe toxicity (8.20 vs. 5.48 µg/ml, *p = 0.015*). No significant differences were observed in C_trough_ medians in other subgroups with and without severe hepatotoxicity (Table 6).

**Table 6:** Impact of Gender and Hepatitis C Virus Co-Infection on Nevirapine Concentrations (mg/ml) in Patients Experiencing Severe Hepatotoxicity.

	Severe Hepatotoxicity	Without Severe Hepatotoxicity	*p*
C_trough_ -median (IQR)	5.91 (4.45, 7.60), n = 32	5.54 (4.33, 740), n = 120	0.290
Gender			
Male	8.20 (5.07, 11.20), n = 13	5.48 (4.31, 7.21), n = 62	0.015
Female	5.43 (4.00, 6.90), n = 19	5.56 (4.41, 7.53), n = 58	0.543
HCV Antibody			
Positive	7.80(4.50, 10.22), n = 13	6.13(4.16, 7.93), n = 37	0.157
Negative	5.48(4.00, 6.90), n = 19	5.21(6.39, 7.00), n = 83	0.884
C_2_ -median (IQR)	6.06(4.63, 7.48), n = 5	6.29(5.20, 7.77), n = 29	0.715

Ctrough: trough concentrations; C2: concentrations at 2 h post-dose; HCV: hepatitis C virus; ALT: alanine aminotransferase. Two patient groups, with and without severe hepatotoxicity, were included for analysis.

Baseline CD4+ T cell count was not statistically associated with the incidence of severe liver toxicity among female Chinese patients, 29% (9/31) and 16% (10/63) in the patients with the baseline CD4+ T count >250/mm^3^, and ≤250/mm^3^, respectively (*p = 0.184*). The hepatitis C virus co-infection rate was 29% (56/191) in our study population. Active HCV co-infection, defined as HCV RNA >10^3^copies/µl, led to a significantly higher incidence of liver toxicity, 44% (10/23) compared to 18% (6/33) among patients without active HCV co-infection (*p = 0.039*, Table 7).

**Table 7:** Risk of Severe Hepatotoxicity in Patients with Different Baseline CD4 Count and Hepatitis C Virus RNA Level.

	Group – n (%)		*p*
	Severe hepatotoxicity	Without Severe Hepatotoxicity	
**Baseline CD4 count (females)**			
>250/mm3	9 (29)	22 (71)	0.184
≤250/mm3	10 (16)	53 (84)	
**HCV RNA level**			
>10^3^copies/ul	10 (43)	13 (57)	0.039
≤10^3^copies/ul	6 (18)	27 (82)	

## Discussion

This study found a significant correlation between nevirapine trough concentrations, virologic response and liver toxicity among Chinese HIV-infected patients. While patients with low nevirapine C_trough_ have a significantly higher risk of virologic failure, those with a high C_trough_ are at greater risk of severe liver toxicity, especially male patients. Our data suggest that for the optimal virologic outcomes, a trough nevirapine concentration of ≥3.9 µg/ml should be sustained throughout the treatment course for Chinese patients with HIV infection. To our knowledge, this is the first report of an association between nevirapine concentration and clinical outcomes in Chinese patient population. This adds to the evidence supporting the usefulness of therapeutic drug monitoring in optimizing treatment of this population.

The relationship between nevirapine concentrations and virologic response has been explored in previous studies, primarily including Caucasians and African Americans. The INCAS trial suggested that a nevirapine plasma concentration range of 3.45–3.88 µg/ml at week 12 was predictive of virologic success after 52 weeks of therapy [4]. A more recent clinical trial of 74 patients confirmed that patients with virologic success had higher concentrations than those with virologic failure (mean ± SD, 4.55±2.08 vs. 2.57±1.64 µg/ml, p = 0.003) [3]. Our results in Chinese patients with HIV infection are consistent with the previous results obtained from clinical trials on Caucasians and Africans, higher nevirapine trough concentrations were associated with a lower rate of virologic failure. The median levels of C_trough_ among Chinese patients with partial and complete response after a 6-month treatment were significantly higher than that among patients with viral failure (Table 2). The regression analysis revealed a significant linear relationship between C_trough_ and viral load (Figure 2). However, no relationship between C_2_ and treatment outcomes was detected in our study population.

No target trough concentration of nevirapine has been established for treatment success in Chinese patients. Although treatment guidelines recommend various target thresholds for nevirapine trough concentrations, these data are predominately from studies in Caucasian and African American patients, which may not be applicable to patients of Asian descent. Additionally, the current recommendations for nevirapine C_trough_ thresholds are not completely in agreement. For instance, an observational cohort analysis [2] suggested 3.0 µg/ml as the minimal C_trough_ because risk of virologic failure in patients with nevirapine concentrations ≤3 µg/ml was significantly increased (relative risk 5.0, 95% CI 1.8–13.7). Another study of 178 patients identified a mean plasma concentration of 4.3 µg/ml to be an independent factor for virologic failure [14]. Although the cut-off of 3.0 µg/ml was tentatively adapted to clinical practice in China according to the 2006 Chinese HIV Treatment Guidelines, this level does ensure treatment success as virologic failures often occur among patients with concentrations >3.0 µg/ml. This phenomenon is possibly explained by racial or ethnicity variability in the effective concentration needed to ensure therapeutic success and an underrepresentation of Chinese patients in previous studies. According to our data, the currently accepted threshold for nevirapine C_trough_ (3 µg/ml) had an unfavorable sensitivity (40%) and may lead to a high incidence of virologic failure. The sensitivity would be improved to 60% without a marked decrease of specificity using 3.9 µg/ml. Therefore, to minimize the risk of virologic failure, we believe a more suitable nevirapine C_trough_ threshold is 3.9 µg/ml in Chinese patients, a 30% increase from the current nevirapine C_trough_ recommendation (3 µg/ml).

Liver toxicity is the most common adverse effect associated with nevirapine treatment while results from previous studies on the relationship of nevirapine C_trough_ and liver toxicity are not in agreement. González de Requena *et al* [6] investigated the effect of nevirapine plasma exposure on liver enzyme elevations and observed that among patients with chronic hepatitis C co-infection, nevirapine concentrations >6 µg/ml were associated with a 92% risk of liver toxicity. Therefore, monitoring nevirapine levels, especially in individuals with chronic hepatitis C co-infection, might be warranted. A multivariate linear regression analysis [15] also revealed that high C_trough_ at week 24 was an independent predictor for liver enzyme elevations. However, a prospective population pharmacokinetic study [5] and the 2NN study [11] suggested no correlation between nevirapine trough concentrations and liver enzyme levels, after corrected for known covariates such as gender, CD4 cell count at baseline, and hepatitis C coinfection.

Our data confirmed no significant differences in nevirapine C_trough_ or C_2_ between patients with and without severe liver toxicity. Several confounding factors such as gender, HCV co-infection and baseline liver function have been suggested for nevirapine exposure and liver toxicity [16], [17], although no association between those factors and the occurrence of severe liver toxicity were detected in our patients. The nevirapine C_trough_ was significantly higher in Chinese male patients with severe liver toxicity. However, such relationship was not observed in females and other subgroups, suggesting the nevirapine-related liver toxicity might be gender dependent. While previous studies [17], [18] have demonstrated that female gender combined with high CD4+ T cell count significantly increased the incidence of nevirapine-induced liver toxicity, our data found a non-significant, modest increase of liver toxicity in female patients with high baseline CD4+ count (>250/mm^3^) (29% *vs.* 16%, p = 0.184). This may be due to the relatively small sample size, along with racial and ethnic factors.

Our data confirmed that hepatitis C virus co-infection was significantly associated with an increased risk of severe liver toxicity in Chinese HIV+ patients [19]. A prospective study [16] of 568 patients, primarily Caucasians and African Americans, also demonstrated a higher risk of severe hepatotoxicity among patients with chronic viral hepatitis C and B infections. However, such risk was remarkably reduced by concurrent interferon-based therapy with sustained HCV clearance in patients with HIV/HCV coinfection [20]. In the present study, Chinese HIV+ patients with high HCV RNA levels had a significantly greater risk of severe liver toxicity than those patients with low HCV RNA levels. This suggests that active HCV coinfection, correlated with HCV RNA levels, might be a more accurate predictor of nevirapine related liver toxicity than positive HCV antibody alone. Although the underlying mechanisms of this hepatotoxicity are not fully understood, an association between increased HCV-specific immune response and T cell activation have been suggested in previous studies [21]. Therefore, based on our results, nevirapine containing regimens should be avoided in Chinese patients with active HCV coinfection until sustained control of HCV is achieved.

The use of nevirapine peak concentration as a monitoring parameter was explored in this study, as limited information is available on this topic to date. Nevirapine C_2_ was chosen to represent nevirapine peak concentration, based on previous pharmacokinetic results that indicate the mean time to reach nevirapine peak concentration was 2.01 hours [22]. Unfortunately, nevirapine C_2_ had no correlation with any clinical outcomes evaluated in this study. This may be due to inter-patient variability, *i.e.* the exact peak time for nevirapine is different from patient to patient. Thus, it is difficult to collect samples that truly represent peak concentrations. Our data suggest that the clinical use of nevirapine peak concentrations may not be practical. Future studies using other metrics, such as area under the time-concentration curve derived from population analysis, are warranted to explore the relationship between pharmacokinetics of nevirapine and clinical outcomes in Chinese HIV+ patients.

There were several limitations to this multi-centre prospective study in patients receiving nevirapine-containing therapy. The cohort was relatively small and heterogeneous, including patients with various underlying medical conditions. However, our patients reflected a typical Chinese HIV+ clinical cohort [1], making the results applicable to the study population. Selection bias was prevented by patient randomization. Because only trough and 2-hour post dose concentrations were included in our study, other untested pharmacokinetic metrics including exposure and clearance may be of more clinical relevance. The strength of some of the associations presented in this study should be interpreted with caution, owing to the relatively small sample size. Finally, unrecognized confounders may have influenced nevirapine concentrations, *e.g.* dosing in relation to food, concurrent medications, or genetic polymorphisms of CYP2B6, which significantly influenced nevirapine metabolism in HIV-infected patients in Uganda [19]. However, genotyping was not routinely available, and dose adjustments based on results of genotypic tests have not been proposed as yet for nevirapine.

In conclusion, this study supports the association between nevirapine concentrations and treatment outcomes in Chinese HIV+ patients. For patients with low trough concentrations, dose adjustments should be considered to achieve and sustain a median nevirapine trough concentration greater than 3.9 µg/ml. Given the inter-patient variability and the lack of an upper pharmacokinetic threshold for nevirapine associated hepatotoxicity, dose modification should be considered only in symptomatic patients. Finally, further studies are warranted, as a therapeutic range for nevirapine in Chinese HIV+ patients remains elusive.

## Methods

### Study subjects

A prospective, randomized study was conducted among antiretroviral-naïve HIV-infected patients of the Han nationality in China. The study protocol was in accordance with the Declaration of Helsinki and approved by the Ethics Committee of participating hospitals including Peking Union Medical College Hospital, Beijing Youan Hospital, Beijing Ditan Hospital, The First Affiliated Hospital, Henan Medical University, Xian Tangdu Hospital, The Second Affiliated Hospital, Xiangya Medical University, Shanghai Public Health Center, Shenzhen CDC, The 8^th^ Hospital of Guangzhou, HIV/AIDS Care Center of Yunnan, and Zhejiang Medical University. Written consent was obtained from each patient. Eligible patients were aged from18 to 65 years, and received a standard regimen that included nevirapine and two nucleoside reverse transcriptase inhibitors between January 2002 and November 2008 from thirteen HIV/AIDS Clinical Centers in China. A total of 227 patients were enrolled in this study (Figure 1). The original study design was published previously [1].

Nevirapine (Desano Pharma, Shanghai, China) was administered at 200 mg once daily for 2 weeks and 200 mg twice daily thereafter. The bioequivalence of nevirapine in reference to Viramune® from Boehringer Ingelheim was demonstrated in the previous studies [23]. Medication adherence was monitored by self-report using validated surveys and directly observed therapy (DOT). Main exclusion criteria were acute HIV infection, AIDS-defining illness within 2 weeks of entry, white blood cell count <2.0×10^9^/L, absolute neutrophil count <1.0×10^9^/L, hemoglobin level <90 g/l, platelet count <0.75×10^12^/L, transaminase and alkaline phosphatase level >3 times the upper limit of the normal range, bilirubin level >2.5 times the upper limit of the normal range, and serum creatinine level >1.5 times the upper limit of the normal range. Patients were followed up prior to starting therapy, then 1, 2, 3, 6, 9 and 12 months after the initiation of treatment. During all of the visits, the clinical assessment was performed and blood samples were taken for liver function and plasma viral load analyses.

### Outcome measures

The primary efficacy outcome was virologic failure, defined as a single plasma HIV-1 RNA concentration (viral load) >400 copies/ml at week 24. Complete virologic response was defined as a viral load <50 copies/ml at week 24, whereas partial virologic response was defined as a viral load between 50 and 400 copies/ml at week 24. The QUANTIPLEX HIV-1 RNA assay was used to measure the plasma HIV-1 RNA concentrations. The limits of detection of the assay, as indicated by the manufacturer, was 50∼500,000 copies/ml.

All clinical adverse events and laboratory abnormal results were documented. Severe liver toxicity was defined as laboratory abnormalities greater than grade 3 (WHO, 1979), including a significant increase (>5 times of normal upper limit) of aspartate aminotransferase (AST), alanine aminotransferase (ALT) or total bilirubin, severe clinical symptoms consistent with liver toxicity, or a therapy change caused by liver toxicity during the first 12 months of treatment.

### Plasma nevirapine concentration analyses

A plasma sample was collected at 2 h after oral administration of nevirapine (C_2_) during DOT or prior to the morning dose for the detection of trough concentration (C_trough_, at least 12-h postdose). The nevirapine concentration in plasma was determined by a validated HPLC assay modified from a previous study [24]. The nevirapine concentration were analyzed on a Shim-pack CLC-ODS column (6 mmID×15 cm, 5 µm) with a mobile phase consisting of water-acetonitrile (23∶77) at a flow rate of 1 ml/min, and the wavelength for detection was 260 nm. Tegafur was used as an internal standard. The calibration curve of nevirapine was linear in the range of 0.05–10 µg/ml (r = 0.9999), and the limit of detection was 0.05 µg/ml. The RSDs of intra- and inter- run validations were less than 7%. The mean recoveries fell in the range of 90–110% for the high, middle and low concentrations. All plasma samples collected at the 6^th^ month of nevirapine treatment were assayed for concentrations. Additional plasma samples were collected for concentration assessment if severe liver toxicity presented. For patients without severe liver toxicity, the nevirapine plasma concentrations were determined during the 12-month follow-up visit.

### Statistical analyses

All statistical analyses were performed using the SPSS 11.5 statistical package. Categorical variables were tested with Chi-Square or Fisher's exact test, and continuous variables were tested with Kruskal-Wallis or student *t*-test. The cut-off value for the effective range of plasma drug concentration was determined by the receiver operating characteristic curve (ROC curve). For all tests, p<0.05 was considered to be statistically significant.
